# Deep learning-driven automatic counting of petal number in cut chrysanthemum inflorescence

**DOI:** 10.1016/j.plaphe.2026.100238

**Published:** 2026-06-11

**Authors:** Shanpeng Xu, Jingshan Lu, Lian Ding, Fei Zhang, Fadi Chen, Zhenxing Wang, Sumei Chen, Weimin Fang, Zhiyong Guan

**Affiliations:** State Key Laboratory of Crop Genetics & Germplasm Enhancement and Utilization, Key Laboratory of Landscaping, Ministry of Agriculture and Rural Affairs, Key Laboratory of Biology of Ornamental Plants in East China, National Forestry and Grassland Administration, College of Horticulture, Nanjing Agricultural University, Nanjing, Jiangsu, 210095, China

**Keywords:** Cut chrysanthemum, Petal number, Deep learning, Machine learning, Image processing

## Abstract

The number of petals in an inflorescence is an important phenotypic indicator for quality evaluation and cultivar identification of cut chrysanthemums (*Chrysanthemum morifolium* Ramat.). Current manual measurement methods are time-consuming, error-prone, and poorly suited to the complex geometry of chrysanthemum flowers, which limits their utility for large-scale phenotyping and breeding programs. Although image-based phenotyping has advanced rapidly, automated and reliable methods for petal counting in densely packed or partially obscured inflorescences remain underdeveloped. Here, we developed a deep learning-based framework for automatic extraction of petal number in cut chrysanthemums. Images from multiple varieties were collected to construct a representative dataset, and petal density maps were generated through manual annotation with Gaussian kernel function. We employed a Congested Scene Recognition Network (CSRNet) enhanced with a Squeeze-and-Excitation (SE) channel attention mechanism (SE-CSRNet) for petal density estimation. Spearman correlation analysis revealed strong agreement between visible and actual petal counts (Spearman's r = 0.953, p < 0.0001). Compared with the original CSRNet, SE-CSRNet reduced mean absolute error (MAE) and root mean squared error (RMSE) by 5.2% and 7.4%, respectively. Further optimization using regression fitting revealed that random forest achieved the best performance (MAE = 4.24, RMSE = 5.06, R^2^ = 0.967), indicating reliable stability and satisfactory generalization under the conditions evaluated in this work. Application of the optimized model to two cut chrysanthemum varieties confirmed its practicality by successfully detecting reductions in petal number under high-temperature stress. Our results demonstrate that integrating dataset construction, deep learning–based density estimation, and machine learning optimization enables efficient and accurate prediction of petal number in cut chrysanthemums.

## 1 Introduction

Petals, as the most conspicuous component of the inflorescence, play a crucial role in attracting pollinators and ensuring successful reproduction [1]. Their number and morphology also serve as key taxonomic markers for distinguishing species or varieties [2,3]. At the molecular level, numerous studies have elucidated the genetic and regulatory mechanisms underlying variation in petal number [4,5]. In ornamental horticulture, petal morphology, number, and arrangement are fundamental criteria for cultivar classification, and the ornamental value of specific types largely depends on the presence of a sufficient number of petals [[6], [7], [8]]. For edible flowers, petal number directly affects yield [9]. Furthermore, cultivars with more petals per inflorescence generally exhibit longer vase life and higher consumer appeal [10]. However, petal number is strongly affected by environmental factors such as light and temperature, which can reduce petal counts or increase deformities, thereby diminishing visual quality [11,12]. Fertilization, in contrast, can increase petal production [13]. Consequently, accurate petal counting is indispensable for evaluating ornamental quality, breeding, and assessing cultivation practices.

Chrysanthemum (*Chrysanthemum morifolium* Ramat.), one of the world's four major cut flowers, includes tens of thousands of cultivars and enjoys wide consumer popularity [14,15]. Cultivars with numerous petals far outnumber single-petaled cultivars, and high-value ornamental types such as rosette, honeycomb, and ping-pong chrysanthemums often bear 200–600 petals per inflorescence. Therefore, petal number is a central trait in both breeding and production [16]. However, the extremely large petal counts in certain cultivars pose considerable challenges for manual quantification.

With the increasing integration of computer vision and agriculture, automated approaches have shown great promise in plant phenotyping. Inflorescence-level studies have achieved progress in flower detection and counting. For example, Millan et al. applied image analysis and nonlinear models to estimate the number of grape flowers in *Vitis vinifera* L [17]. Wan et al. employed unmanned aerial vehicle (UAV)-based imaging combined with vegetation indices to develop random forest (RF) and optimal subset regression (OSR) models, which were used to predict flower counts in oilseed rape (*Brassica napus* L.) [18]. Farjon et al. used faster region-based convolutional neural network (Faster R-CNN) for apple (*Malus pumila* Mill.) inflorescence estimation [19]. Deep learning has also been used for segmenting and detecting *V. vinifera* inflorescences [20] and for assessing petal damage in roses [21]. Despite these advances, no studies have yet addressed automated petal counting.

Manual counting remains predominant method for determining petal number, but it is labor-intensive, error-prone under complex morphologies, and subject to operator bias. These limitations highlight the urgent need for automated approaches capable of handling densely distributed, morphologically diverse, and small-sized petals. Interestingly, parallel progress in computer vision has been achieved in crowd counting, which faces challenges highly analogous to those of petal counting, including dense distributions, occlusions, and scale variability. Convolutional neural network (CNN)-based crowd counting methods, in particular, have demonstrated high accuracy by learning nonlinear relationships between images and counts [22]. CNN-based crowd counting methods can generally be categorized into major types: object detection-based, density map-based, and point detection-based approaches.

(1) Object detection-based approaches [23,24], such as Faster R-CNN (Faster Region-based Convolutional Neural Network) [25]and YOLO (You Only Look Once) [26], primarily extract image features using CNNs, generate candidate regions, and then localize and classify individuals. The final count is obtained by summing the valid detection boxes. In petal-counting tasks, this strategy requires precise localization of each petal's bounding box. However, overlapping petals, blurred edges, or adhesion to sepals can easily cause missed or duplicate detections, leading to reduced counting accuracy.
(2) Density map-based approaches represent the mainstream solution for dense crowd counting. These models predict a pixel-level density map and integrate it to obtain the total crowd count, effectively mitigating occlusion and scale variation [27]. For example, the Multi-column Convolutional Neural Network (MCNN) employs three sub-networks with different convolutional kernels to extract multi-scale features, but its numerous parameters incur high computational cost [28]. CSRNet, in contrast, adopts VGG16 as a backbone and introduces dilated convolutions to enlarge the receptive field while reducing parameters, generating finer density maps [29]. This approach performs indirect counting, circumventing explicit object detection and offering high robustness in dense scenes. However, its accuracy depends on the sufficiency of feature extraction, and inadequate features for distinguishing overlapping heads may lead to inaccurate density estimation. In petal-counting, head-center annotations are replaced by petal centers, and Gaussian kernels are used to generate petal density maps for model training. The predicted density map is then integrated to estimate total petal number. Nonetheless, diverse petal morphologies and interference from background components (e.g., leaves and sepals) can still introduce prediction errors.
(3) Point detection-based approaches transform the counting task into detecting key points, such as head centers, and derive total counts by localizing these points. The network is trained to predict point coordinates, balancing detection accuracy and adaptability in dense scenarios. Representative models include the Point-supervised Deep Detection Network (PSDDN), which iteratively refines pseudo bounding boxes from point annotations [30], and the Point-to-Point Network, which directly predicts annotated head centers [31]. These approaches feature simple architectures and good performance but require highly discriminative feature learning. In complex scenes with large size variations and cluttered backgrounds, localization errors and threshold sensitivity in peak detection can lead to false or missed detections. For petal counting, “head centers” can similarly be replaced with “petal centers”, and the total count derived from detected points.

From a methodological perspective, object detection-based methods rely on a “bounding-box localization – individual classification – count aggregation” pipeline, with their accuracy heavily dependent on clear boundaries. Point detection-based methods, which rely on precise key-point localization, are sensitive to minor feature variations and threshold selection. Petals, however, exhibit both boundary ambiguity and morphological diversity: overlapping or fused petals hinder accurate bounding-box localization, while subtle or indistinct center features complicate point detection. In contrast, density map-based methods adopt a “Gaussian-kernel annotation – density distribution learning – integral counting” process. By learning the mapping between petal pixels and density values rather than explicit boundaries or centers, they inherently avoid the influence of morphological irregularities, making them more suitable for petal-count estimation.

Among density map-based methods, CSRNet stands out as an end-to-end CNN model designed to address scale variation, severe occlusion, and high object density in complex counting scenarios. By integrating dilated convolutions into the VGG16 backbone, CSRNet enlarges the receptive field without sacrificing spatial resolution, alleviating parameter redundancy while enhancing multi-scale feature extraction. This architectural advantage aligns well with petal-counting tasks, which involve significant scale variability among petals.

Moreover, attention mechanisms have proven effective in improving feature extraction in CNN-based architectures and are widely employed in intelligent agricultural phenotyping to capture subtle morphological or physiological traits. Among them, the Squeeze-and-Excitation (SE) attention mechanism [32], has gained prominence for its lightweight and efficient design. Through global average pooling, two fully connected layers, and a Sigmoid activation, SE recalibrates channel-wise feature responses with minimal computational overhead, enhancing model representational capacity without compromising real-time performance—an essential requirement for practical phenotyping applications.

Therefore, to address the challenges posed by petal overlap, morphological diversity, and background interference, this study proposes a phenomics-oriented deep learning framework for estimating petal numbers in cut chrysanthemums. This method integrates biological validation, trait-specific optimization, and statistical calibration to support reliable floral phenotypic analysis.

First, images of multiple cut chrysanthemum cultivars were collected to construct a petal-counting dataset, and petal density maps were generated through Gaussian-kernel-based manual annotation. A density-map regression model based on CSRNet was then employed to estimate petal distribution in high-density inflorescences. To better address petal-specific visual characteristics such as color similarity, dense arrangement, and channel redundancy, the SE channel attention mechanism was incorporated to enhance feature representation and improve sensitivity to subtle petal textures.

Second, considering the discrepancy between the number of visible petals in the images and the actual biological number of petals, we introduced a correlation-based validation step. Statistical analysis was performed to verify the relationship between image-visible petal counts and actual biological counts, providing biological justification for using image-based density estimation as a phenotyping proxy.

Third, to improve prediction robustness under high-density conditions, a regression-based calibration step was applied to the CSRNet outputs. By combining deep learning-based density estimation with a machine-learning regression model, a two-stage hybrid prediction framework was constructed to reduce nonlinear deviations and enhance prediction stability.

Finally, the estimated petal numbers were integrated into a statistical analysis pipeline for phenotypic evaluation. By coupling automated petal counting with statistical analysis of phenotypic traits, the proposed framework enables quantitative assessment of floral responses under environmental stress, providing a practical tool for high-throughput phenotyping and breeding research in chrysanthemum.

Overall, this study establishes an integrated methodological framework that combines density-map-based deep learning, biological validation, and calibration-enhanced prediction to enable accurate quantification of dense floral organs. The proposed approach provides a feasible technical pathway for automated inflorescence phenotyping and intelligent breeding in floricultural research.

## 2 Materials and methods

**Fig. 1:**
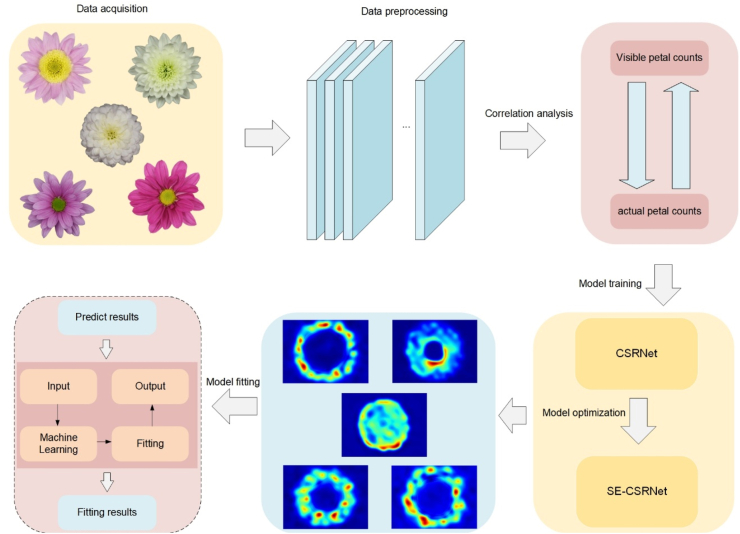
Technology roadmaps. Schematic workflow of the deep learning-based framework for automated flower petal counting, encompassing data acquisition, preprocessing, model training with CSRNet and SE-CSRNet, and result prediction.

The technical workflow of this study is illustrated in Fig. 1.

### 2.1 Data acquisition and dataset preparation

#### 2.1.1 Data acquisition

The experimental images were collected at the Baguazhou Chrysanthemum Pilot Plant Base (Nanjing Agricultural University, Qixia District, Nanjing, Jiangsu Province; 118°50′21.336″ E, 32°12′57.233″ N) and the Hushu Chrysanthemum Research and Development Base (Nanjing Agricultural University, Jiangning District, Nanjing; 118°56′18.092″ E, 31°48′19.904″ N). Data collection was conducted from October 31 to November 20, 2018. Cut flowers were transported to the photography studio immediately after harvest. Images were captured with a Canon EOS 5D Mark II RGB camera (Canon Inc., Tokyo, Japan) equipped with an EF24–105 mm f/4 L IS USM lens. The camera was mounted on a tripod positioned at the center of a photography box, with the lens oriented perpendicular to its top surface. To capture the floral morphology, stems were removed, leaving only the inflorescences, which were positioned flat on a screen. The collected varieties include 90 introduced varieties such as ‘Vatican’, ‘Rossi’, and ‘Cinzia’, and 193 self-developed varieties including ‘Nannong Hongyun’, ‘Nannong Sajin’, and ‘Nannong Hongque’, covering single-headed cut chrysanthemum varieties and multi-headed cut chrysanthemum varieties. Variety name information is shown in Supplementary Table S1. Therefore, the data distribution is universal and the subsequent recognition model is highly robust. A schematic diagram of the data collection process is presented in Supplementary Fig. S1.

#### 2.1.2 Dataset preparation

The preparation of the experimental data set includes steps such as data screening and annotation, and density map preparation.

(1) Data screening: The collected cut chrysanthemum image data is preliminarily screened to remove images with poor quality such as blur and high similarity. After screening, a total of 694 chrysanthemum photos were collected, which still include all the varieties mentioned above. To minimize cultivar-related bias, the sample distribution across cultivars was examined prior to model training. Most cultivars were represented by 2–3 images, and no single cultivar dominated the dataset.
(2) Data annotation: The filtered images were manually annotated using the open source annotation software Labelme. Each petal was annotated with the category “petal”. The annotated file was exported to JSON format, which contains the coordinate information of each detection target position, target category and target real point.
(3) Density map preparation: In order to adapt to the training requirements of the crowd counting model, the annotated JSON file needs to be further converted into the MAT format that is easy for the model to read, and the corresponding petal density map is generated. The density map is the core supervisory signal for the counting task. Its essence is to convert the discrete petal annotation points in the image into a continuous density distribution map so that the integral value of all pixels on the density map is equal to the actual number of petals. Density maps were generated using a geometry-adaptive Gaussian kernel strategy. Specifically, a delta function was placed at each annotated point and subsequently convolved with a Gaussian filter. The standard deviation of the Gaussian kernel was adaptively determined according to the local distribution density of neighboring annotations. For each annotation point, the distances to its second, third, and fourth nearest neighboring points were computed using a KD-tree, and the average local neighbor distance was used to determine the Gaussian spread. Following the implementation in our study, the standard deviation was set as 0.1 times the sum of these neighboring distances. For images containing only one annotation point, the standard deviation was set to one-quarter of the average image dimension. The Gaussian kernel size was not manually fixed but automatically determined by the scipy. ndimage.gaussian_filter function according to the corresponding standard deviation. The flowchart for dataset preparation is shown in Supplementary Fig. S2.
(4) Dataset Partitioning: We randomly partition the labeled images into a training set, validation set, and test set ratio of 7:2:1. This dataset partitioning strategy is designed to ensure that the model is fully validated and tested during the training process, thereby ensuring the performance stability and reliability of the model in practical applications. During dataset splitting, images from different cultivars were randomly distributed across the training, validation, and test sets to maintain cultivar diversity.

#### 2.1.3 Correlation analysis

In studying chrysanthemum inflorescence petal counts using the CSRNet algorithm, image acquisition was influenced by factors such as the natural growth morphology of inflorescences (e.g., petal stacking), shooting angle, and environmental occlusion. Consequently, partial petal occlusion occurred, meaning that the visible petals captured in the image represented only a subset of the total petal count. Some petals were not visible in the images because they were either occluded by upper petals or positioned in blind spots, leading to a natural discrepancy between the visible and actual petal counts.

Relying solely on visible petal features risks prediction errors due to incomplete data representation. Therefore, it was necessary to verify the statistical correlation between visible and actual petal counts to support the rationale of the CSRNet prediction framework. The analysis process and verification objectives were as follows.

(1) Sample selection: The experimental sample set included chrysanthemum inflorescences of different varieties, flower shapes, and varying degrees of occlusion to ensure representativeness.
(2) Variable definition and data collection: Independent variable (X): The number of visible petals in the image, obtained through manual annotation. Each sample image was annotated three times, and the average value was taken to reduce counting errors. Dependent variable (Y): The actual number of petals per inflorescence. Each sample was fully peeled and manually counted, repeated three times with the average value recorded to ensure accuracy.
(3) Correlation analysis: The analysis employed both Pearson and Spearman correlation coefficients. If the distributions of X (visible petals) and Y (actual petals) met normality assumptions (tested by the Shapiro–Wilk test), the Pearson coefficient quantified linear correlation strength [33]. Otherwise, the Spearman rank coefficient was applied to assess monotonic correlation [34]. A hypothesis test (α = 0.05) was conducted to determine statistical significance, thereby testing whether the number of visible petals was significantly correlated with the actual petal count [35].
(4) Verification significance: If the correlation coefficient between visible and actual petal counts was ≥0.7 (indicating strong correlation) with p < 0.05 (statistical significance), this implied that despite occlusion, CSRNet could accurately predict actual petal counts by learning this statistical relationship, thus supporting the reliability of its predictions. Conversely, if the correlation was weak, further optimization of the sample collection process (e.g., adjusting shooting angles, adding multi-view fusion) or enhancing model input features (e.g., introducing feature-completion modules for occluded regions) would be required to improve prediction accuracy.

### 2.2 Petal counting algorithm

#### 2.2.1 CSRNet

Extracting the number of petals in cut chrysanthemums facilitates variety identification and quality assessment. Therefore, counting accuracy is particularly important. Given the potential application scenario within large-scale production bases, the detection model should possess the following characteristics: good detection accuracy, speed, and portability. Based on comprehensive considerations, this paper selects the CSRNet algorithm as the petal number extraction model.

In terms of network architecture, CSRNet primarily consists of two components: a front-end feature extraction module and a back-end density map regression module. The front-end feature extraction module uses the first 10 layers of a pre-trained VGG16 network as the backbone. This module fully extracts both deep semantic features and shallow detail features from the input image, laying a solid feature foundation for subsequent density map generation. Notably, the back-end density map regression module innovatively incorporates the dilated convolution operation. By introducing a dilation rate into the convolution kernel, dilated convolution effectively expands the receptive field of the convolution operation without increasing network parameters or computational overhead, thereby better capturing the spatial distribution of objects of varying scales within the image. Specifically, following the original CSRNet design, the back-end module stacks six dilated convolutional layers with a dilation rate of 2 to enlarge the receptive field and aggregate contextual information while maintaining the spatial resolution of the feature maps extracted by the front-end. Ultimately, it generates a density map with precise spatial location information. By integrating this density map, the object count result is obtained. In terms of loss function design, CSRNet uses a mean squared error loss function to optimize the difference between the predicted and true density maps, achieving an end-to-end training process and avoiding the limitations of traditional methods that rely on manually engineered features.

CSRNet has demonstrated strong suitability for dense object counting tasks and was selected as the foundational model in this study based on task-specific and application-oriented considerations.

First, in terms of structural compatibility with chrysanthemum inflorescences, petals are densely arranged on the surface of the flower head, with frequent mutual occlusion and moderate scale variation between central and peripheral regions. These characteristics resemble high-density counting scenarios, where spatial resolution preservation and contextual aggregation are critical. CSRNet employs dilated convolutions to enlarge the receptive field without reducing feature map resolution, enabling effective capture of overlapping structures while retaining fine-grained spatial details. This property is particularly advantageous for petal density estimation, where precise localization of density distribution is more important than explicit instance detection.

Second, although more recent crowd-counting models (e.g., those incorporating multi-path feature fusion, dynamic density generation, or Transformer-based attention mechanisms) [[36], [37], [38], [39]] have achieved improved benchmark performance, these methods often introduce substantially higher computational complexity and parameter counts. Their core optimizations are typically designed to address extreme perspective variation and scale disparities (e.g., tiny distant objects versus large foreground instances), which are less pronounced in near-orthographic chrysanthemum imaging conditions. In our task, scale differences among petals are present but do not reach the extreme levels observed in crowd scenes. Therefore, adopting highly complex architectures may introduce redundant modules and unnecessary computational burden without proportional performance gain.

In contrast, CSRNet provides a balanced trade-off between accuracy, computational efficiency, and structural simplicity. Its moderate parameter size facilitates stable training on medium-scale agricultural datasets and enables potential deployment on embedded or mobile devices, which aligns with the practical requirements of real-time field phenotyping.

Therefore, considering structural suitability, computational efficiency, and application feasibility, CSRNet was selected as the core density estimation model for petal number extraction in cut chrysanthemums. A schematic diagram of the CSRNet network structure is shown in Supplementary Fig. S3.

#### 2.2.2 Add SE attention module

The Squeeze-and-Excitation (SE) attention module is a lightweight and efficient channel-wise attention mechanism. Its core concept is to improve the feature representation capability of convolutional neural networks by modeling the interdependencies between feature channels and adaptively adjusting the weights of each channel's features. Traditional convolution operations uniformly process the spatial and channel dimensions of input features, failing to explicitly address differences in importance between channels. The SE module effectively addresses this issue by explicitly modeling channel relationships. The SE module consists of two main steps: Squeeze and Excitation. In the Squeeze phase, global average pooling is used to compress the input feature map, aggregating the spatial information of each channel into a global description vector to capture global context. In the Excitation phase, a gating mechanism consisting of a fully connected layer and a nonlinear activation function models the correlations between channels and generates a set of normalized weight coefficients. Finally, the SE module multiplies these weight coefficients with the original features channel by channel, enhancing important feature channels and suppressing redundant ones. Compared to traditional convolutional neural networks, the SE module can effectively improve the network's discriminative capabilities without significantly increasing computational complexity or parameter count. The SE attention module was inserted after the VGG16 frontend feature extractor and before the dilated convolution-based backend network. Specifically, the frontend network adopted a VGG16-based feature extractor, whose output feature map contained 512 channels. The SE module first applied global average pooling to compress the spatial dimensions and generate channel-wise descriptors. Subsequently, two successive 1×1 convolution layers were used to model inter-channel dependencies, where the channel dimension was reduced from 512 to 32 using a reduction ratio of 16, followed by restoration back to 512 channels. ReLU and Sigmoid activation functions were employed after the first and second convolution layers, respectively. Finally, the generated channel attention weights were multiplied with the original feature map to recalibrate channel responses adaptively. The recalibrated features were then fed into the backend network for density map regression. The structure of the SE attention mechanism module is shown in Supplementary Fig. S4 below.

To evaluate the impact of different backbone networks on model performance, this study incorporated several commonly used attention modules, including Coordinate Attention (CA) [40], Efficient Channel Attention (ECA) [41], Global Attention Mechanism (GAM) [42], and Simple Parameter-Free Attention Module (SimAM) [43]. These modules were individually integrated into the model to identify the backbone that achieves the highest accuracy.

### 2.3 Machine learning model fitting

To further enhance the accuracy of petal number prediction, we employed machine learning models to fit and analyze the results generated by CSRNet. Specifically, five commonly used regression models were evaluated: random forest regression [44], lasso regression [45], linear regression [46], support vector regression (SVR) [47], and k-nearest neighbors (kNN) regression [48]. By fitting, validating, and comparing these models, we identified the most suitable approach for petal number prediction, thereby improving the overall prediction accuracy.

To assess the performance and robustness of the regression models, four statistical metrics were employed: MSE, which measures the average of the squared differences between predicted and actual values; RMSE, the square root of MSE, representing the average prediction error; MAE, the average of the absolute differences between predicted and actual values; and R^2^, which reflects the proportion of variance explained by the model, with values closer to 1 indicating a better fit.

### 2.4 Model training

#### 2.4.1 Training platform

The host parameters used in this study are shown in Supplementary Table S2.

The training was conducted using the PyTorch 2.4.0 deep learning framework, with Python 3.10 and CUDA 12.4 configured on a Windows 11 operating system.

#### 2.4.2 Model training parameters

The unified training parameters were configured as follows: a batch size of 1, 200 training epochs, an initial learning rate of 1e-5, a weight decay coefficient of 5e-4, stochastic gradient descent (SGD) as the optimizer, and a momentum value of 0.95.

#### 2.4.3 Evaluation metrics

The performance of the CSRNet counting model was evaluated using two statistical metrics: MAE and RMSE.

### 2.5 Model application analysis

High temperatures can substantially affect the growth and development of cut chrysanthemums, often resulting in a reduced petal number and, consequently, decreased commercial value. To investigate this effect, the ‘Huang Ping Pang’ and ‘Yuka’ varieties were selected as experimental materials. Plants exhibiting growth abnormalities (e.g., pest or disease damage) were excluded, and samples were collected from both the natural flowering period (control) and from plants subjected to high-temperature stress. The two groups differed in planting date, which resulted in different temperature regimes during the vegetative and reproductive phases:

High-temperature treatment group: Plants were transplanted on July 1, 2024, thus experiencing the highest naturally occurring temperatures during the vegetative and early bud development stages (July–August 2024). Control group: Plants were transplanted on August 1, 2024, and flowered under relatively milder late-season temperatures.

The temperature conditions during July–August 2024 at the experimental site (Baguazhou Chrysanthemum Pilot Plant Base, Nanjing, China; 118°50′21.336″ E, 32°12′57.233″ N) were shown in Supplementary Fig. S5. The daily mean temperature trend shows that peak temperatures were concentrated from late July to mid-August (July 21 – August 12), confirming that plants transplanted on July 1 experienced significantly higher temperatures during the early growth period compared with those transplanted in August (there were 17 days with daytime maximum temperatures ≥35 °C and 22 days with nighttime maximum temperatures ≥30 °C). Accordingly, the July-transplanted plants were designated as the high-temperature stress group, while the August-transplanted plants served as the natural temperature control group.

Plants were grown under identical agronomic management except for the naturally occurring temperature differences associated with planting date. Rom each plant, one primary inflorescence was selected for analysis, yielding 58 images for the control group and 58 images for the high-temperature treatment group. The images were taken between October 16, 2024 and November 15, 2024. These images were input into the trained SE-CSRNet model to predict the petal number for each inflorescence.

The predicted petal numbers were then statistically analyzed. For both groups, the mean, standard deviation, and coefficient of variation of petal number were calculated. In addition, an independent-samples *t*-test (significance level α = 0.05) was conducted to evaluate whether the differences in petal number between the control and high-temperature groups were statistically significant, thereby quantifying the impact of high-temperature treatment on chrysanthemum petal number.

## 3 Results and analysis

### 3.1 Correlation analysis

A correlation analysis was conducted between the number of visible petals and the actual number of petals in chrysanthemum inflorescence images to assess the feasibility of predicting the true petal count based on image features. The analysis incorporated both the Shapiro-Wilk normality test and Spearman correlation analysis. The specific results are presented below.

#### 3.1.1 Data distribution and suitability of analytical methods

The Shapiro-Wilk normality test was applied to evaluate the distribution characteristics of the visible petal count and the actual petal count. The results indicated that neither variable followed a normal distribution (p < 0.05). Consequently, the Spearman rank correlation method was selected, as it does not require the normality assumption and can effectively capture monotonic relationships between the two variables. This approach is well-suited to the data characteristics and analytical requirements of this study.

#### 3.1.2 Correlation strength and statistical significance

Spearman correlation analysis revealed a strong positive correlation between the number of visible petals and the actual number of petals, with a correlation coefficient of r = 0.953 and a significance level of p < 0.0001. This indicates that, despite partial occlusion of petals in the images, changes in the visible petal count closely reflect changes in the actual petal count; as the number of visible petals increases, the actual petal number increases in a consistent and significant manner. Statistically, a p-value below 0.0001 indicates that the probability of this strong correlation arising by chance is less than 0.01%, confirming the reliability and stability of the observed association. The analysis results are illustrated in the Supplementary Fig. S6 below.

In summary, the strong positive correlation between the number of visible petals and the actual petal count in chrysanthemum inflorescences provides a robust statistical foundation for employing the CSRNet algorithm to predict actual petal numbers from images. These results validate the feasibility of the proposed approach and establish a basis for subsequent algorithm optimization and practical application.

### 3.2 Model analysis

#### 3.2.1 Comparison of different attention modules

To assess the impact of different attention modules on model performance, the attention modules introduced in Section 2.2.2 were individually integrated into the model. The experimental results are summarized in Supplementary Table S3, which presents the performance metrics for each modified attention module.

The results indicate that the SE-CSRNet model achieves the lowest MAE and RMSE, with reductions of 5.2% and 7.4%, respectively, compared to the original CSRNet model. This improvement minimizes the average deviation in petal counts while effectively mitigating extreme errors in scenarios with dense or occluded petals. The training time for SE-CSRNet was 8.71 h, only 0.16 h longer than that of the original model. These findings demonstrate that the SE attention mechanism enhances model performance without imposing a substantial computational burden, as its channel-wise weighting is achieved solely through global average pooling and lightweight fully connected layers. Consequently, SE-CSRNet provides an effective balance between prediction accuracy and computational efficiency.

#### 3.2.2 Model comparative analysis

**Fig. 2:**
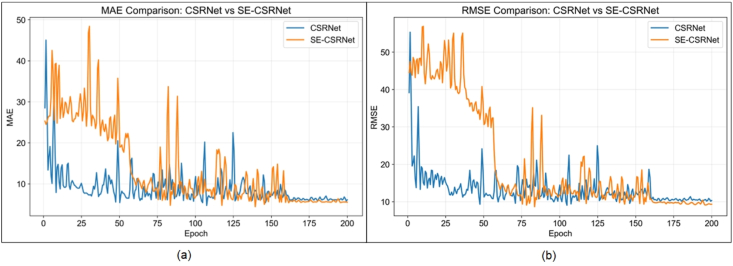
Comparison Chart of MAE and RMSE Curves Before and After Improvement. Performance comparison of CSRNet and SE-CSRNet in terms of (a) Mean Absolute Error (MAE) and (b) Root Mean Square Error (RMSE) across 200 training epochs.

To evaluate the training performance of CSRNet and SE-CSRNet, changes in their MAE and RMSE were monitored throughout the training process. The results are illustrated in Fig. 2.

The analysis shows that, with increasing training epochs, the MAE and RMSE of both models decrease and gradually stabilize. During the early training stages, SE-CSRNet exhibits slightly higher errors than CSRNet due to the additional parameters introduced by the SE module, with fluctuations arising from unstable parameter initialization and gradient updates. In the middle and later stages of training (epochs >50), the channel attention mechanism of the SE module becomes effective, and the errors of SE-CSRNet converge toward or even fall below those of CSRNet. By the later stages (epochs >100), the error curves of both models stabilize at low levels.

Although SE-CSRNet experiences higher initial error due to the additional parameter learning requirements, its channel-wise attention mechanism enhances feature utilization over prolonged training, enabling SE-CSRNet to match or surpass CSRNet in error performance. Both models converge within 200 epochs, and their errors remain stable thereafter, with no signs of overfitting or underfitting. In summary, while incorporating the SE module increases early training complexity, it ultimately improves feature extraction accuracy and error performance, providing valuable insights for optimizing models in similar dense object counting tasks.

To further validate the performance of the proposed algorithm, CSRNet was compared with several mainstream object detection models, including CANNet [49], MCNN, and SASNet [50]. The results are summarized in Supplementary Table S4.

The improved SE-CSRNet outperformed all other algorithms in terms of both MAE and RMSE, demonstrating improved detection accuracy. These results highlight that structural enhancements, such as the incorporation of the SE attention mechanism, can effectively improve prediction performance, providing strong model support for the petal counting task.

To visually evaluate the detection performance, an independent test set accounting for 10% of the total dataset was constructed using a train/validation/test split ratio of 7:2:1. Predictions obtained by the improved model were compared with those of the original CSRNet, with the corresponding results presented in Supplementary Table S5.

**Fig. 3:**
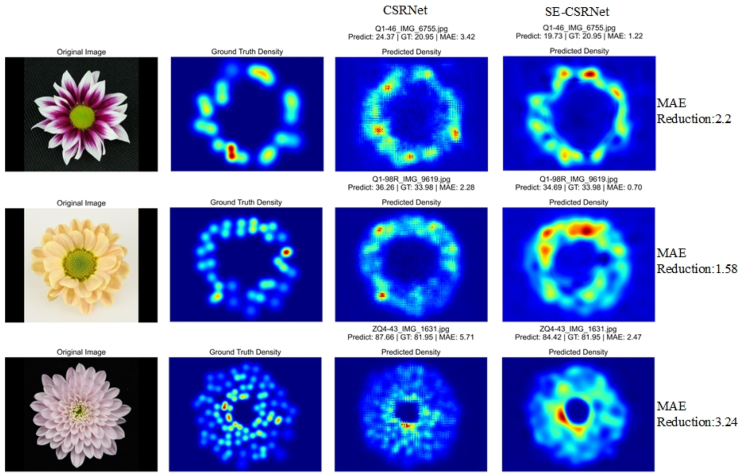
Test result chart. This figure presents three representative test cases, each showing the original flower image, the corresponding density maps predicted by CSRNet and SE-CSRNet, and the ground truth density map. The quantitative metrics (predicted count, ground truth count, and MAE) are provided above each case.

On the test set, SE-CSRNet exhibited improved density estimation performance compared to the original CSRNet. Specifically, SE-CSRNet achieved a mean absolute error (MAE) of 6.0621, lower than CSRNet's 6.7308, indicating higher prediction accuracy. Furthermore, SE-CSRNet substantially reduced the root mean square error (RMSE)—9.3909 versus 12.5178—demonstrating enhanced robustness in mitigating overall error, particularly for extreme cases. These findings confirm that the SE attention mechanism effectively optimizes CSRNet, improving its reliability for density estimation tasks. Furthermore, the computational complexity analysis demonstrated that the performance improvement was achieved with only negligible additional overhead. Compared with CSRNet, SE-CSRNet showed only a slight increase in parameter size (from 16.26 M to 16.29 M), while the increase in GFLOPs was almost negligible (132.2 G). Similarly, the inference speed changed only marginally (17.45 m s/P vs. 17.65 m s/P). Representative examples of test set results before and after the improvement of CSRNet are presented in Fig. 3.

The test results indicate that the improved SE-CSRNet model reduces the MAE by 2.2, 1.58, and 3.24 compared to the original model across different evaluation scenarios. It demonstrates improved prediction accuracy, error mitigation, and density distribution fitting. By incorporating the SE attention mechanism, CSRNet automatically learns channel-wise weights and adaptively adjusts feature representations, enhancing global information integration. When processing chrysanthemum images, the model emphasizes key inflorescence features, accurately capturing density-related information, and producing predictions closer to the true petal counts, thereby achieving improved overall performance.

#### 3.2.3 Machine learning model fitting

By fitting, validating, and evaluating the performance of five common regression models, we designed a model that best suits petal number prediction. The fitting results for each model are shown in Supplementary Table S6 below.

In this study, there is an approximate linear relationship between the predicted value and the actual value after processing by the count model. Linear regression and Lasso regression outperform SVR (with insufficient adaptability of kernel function and parameters) and KNN (susceptible to data distribution, k value, and outliers) because of the obvious intrinsic linear relationship in the data and the simple model that is not prone to overfitting. Random forest becomes the best-performing model due to its ability to reduce variance, capture complex relationships, and be robust to noise through ensemble learning.

### 3.3 Model application analysis

#### 3.3.1 Model testing results analysis

SE-CSRNet was used to count the acquired images of the control and high-temperature treatment groups of the two varieties. The model processing results are shown in Supplementary Table S7 below.

The model processing results are shown in Supplementary Fig. S7 below.

The results showed that due to high temperature stress, some petals developed incompletely, making it difficult for the model to distinguish between normal and abnormal petals. For varieties with fewer petals, the model's predictions remained high, with low errors. However, for varieties with more petals, the model exhibited greater errors, prone to misidentifying abnormal petals as normal, but the errors remained within a manageable range.

Beyond model performance, the observed prediction deviations under high-temperature stress also reflect underlying biological alterations in floral organ development. High temperature is known to interfere with floral meristem activity and organ primordia differentiation, potentially shortening the duration of petal initiation or disrupting normal cell proliferation patterns during early inflorescence development [[51], [52], [53], [54]]. As a result, certain petals may remain morphologically incomplete, reduced in size, or partially fused. Such developmental alterations increase visual heterogeneity within the inflorescence and modify the spatial distribution patterns of petals, which can influence the accuracy of density-map-based petal number estimation. Therefore, the model errors observed under stress conditions may not solely represent computational limitations but could also indirectly reflect stress-induced instability in floral organogenesis. From a phenotyping perspective, such deviations provide additional information on developmental robustness and morphological plasticity under high-temperature stress, a key form of abiotic stress affecting floral development.

#### 3.3.2 Differences in characteristics between the control and high-temperature treatment groups

The mean, standard deviation, and coefficient of variation of petal number were calculated for the control and high-temperature treatment groups of ‘Yuka’ and ‘Huang Ping Pang’ to assess differences in baseline petal number and response to high-temperature stress. The results are summarized in Supplementary Table S8.

Under control conditions, ‘Huang Ping Pang’ exhibited a significantly higher mean petal number (388.43 petals/plant) than ‘Yuka’ (84.45 petals/plant), approximately 4.6 times greater, indicating a substantially higher baseline petal number. The standard deviations were comparable (25.07 for ‘Huang Ping Pang’ and 24.71 for ‘Yuka’), whereas the coefficient of variation for ‘Yuka’ (29.26%) was markedly higher than that for ‘Huang Ping Pang’ (6.45%), reflecting greater inter-individual variability and lower uniformity in ‘Yuka’, while ‘Huang Ping Pang’ displayed a stable and uniform petal number.

Following high-temperature treatment, the petal number decreased significantly in both varieties, by 60.0% in ‘Huang Ping Pang’ and 52.6% in ‘Yuka’, indicating similar relative responses. However, ‘Huang Ping Pang’ experienced a larger absolute reduction, accompanied by increases in standard deviation and coefficient of variation, suggesting reduced uniformity and intensified individual differences. In contrast, ‘Yuka’ exhibited decreased standard deviation and coefficient of variation, indicating increased population uniformity. These results highlight significant differences in the magnitude and stability of responses to high-temperature stress between the two varieties.

Furthermore, the contrasting responses observed between the cultivars ‘Yuka’ and ‘Huang Ping Pang’ suggest cultivar-dependent differences in developmental stability under thermal stress. The significant increase in the coefficient of variation observed in ‘Huang Ping Pang’ indicates reduced phenotypic uniformity and greater developmental instability under elevated temperature conditions. In contrast, the decreased variability observed in ‘Yuka’ may imply a comparatively more stable developmental regulation or a different morphological adjustment strategy under stress.

Importantly, these results demonstrate that automated petal counting should not be viewed solely as a numerical measurement tool, but rather as a means of quantifying morphological variation at the population level. By converting morphological variation into measurable numerical traits, the proposed framework enables high-throughput phenotyping of stress tolerance, offering potential applications in cultivar screening, breeding selection, and climate adaptation research.

#### 3.3.3 Comparison of independent samples *t*-test results and verification of statistical significance

To confirm the statistical reliability of the high-temperature inhibitory effect on petal number in both varieties, an independent samples *t*-test was conducted at a significance level of α = 0.05. Homogeneity of variance was first assessed using Levene's test to determine the appropriate *t*-test type. The results are presented in Supplementary Table S9.

The two cultivars exhibited significant differences in variance homogeneity, reflecting the distinct effects of high-temperature treatment on data dispersion. For ‘Yuka’ Levene's test yielded a statistic of 3.759 with a p-value of 0.067 (p > 0.05), satisfying the homogeneity of variance assumption. This indicates that the dispersion of petal number data between the control and high-temperature groups for ‘Yuka’ did not differ significantly, and therefore a standard *t*-test assuming equal variances was applied. In contrast, for ‘Huang Ping Pang’, Levene's test produced a statistic of 17.885 with p < 0.001, indicating a significant violation of variance homogeneity. This result aligns with the observed substantial increase in data dispersion after high-temperature treatment, and consequently Welch's *t*-test was employed, which adjusts the degrees of freedom to account for unequal variances.

Both cultivars exhibited highly significant differences between the control and high-temperature groups (p < 0.001), with positive t-statistics in both cases, further confirming that high-temperature stress exerts a statistically significant inhibitory effect on petal number.

## 4 Discussion

This study presents an improved approach combining deep learning and machine learning for predicting petal numbers in cut chrysanthemum inflorescences and validates its effectiveness through comprehensive experiments. By applying the CSRNet architecture and further optimizing it with the SE attention mechanism and multiple regression models, the proposed approach demonstrates substantial improvements in accuracy, robustness, and generalization. These findings highlight its potential as a novel and effective tool for automated phenotypic trait extraction in floricultural crops.

### 4.1 Performance of the improved model

This study first verifies the high suitability of the CSRNet model for the petal counting task. Petals are densely arranged and exhibit substantial scale differences (e.g., between central and peripheral petals), which often leads traditional models to miscount due to local feature confusion. CSRNet addresses this challenge through dilated convolutions, which expand the receptive field without reducing resolution, and segmented convolutions, which capture fine-scale features in dense regions. Together, these characteristics allow CSRNet to produce density maps that more closely approximate actual petal distributions. Consistent with its proven effectiveness in crowd counting [[55], [56], [57]]. CSRNet exhibits strong robustness in handling occlusion, scale variation, and petal density. However, the original CSRNet treats all channel features equally, regardless of their relevance. As a result, channels focusing on background noise or irrelevant textures are weighted similarly to those capturing critical petal features, such as edges, textures, and color cues. This limitation reduces the model's discriminative power under challenging conditions, including occlusion and high-density overlaps.

To address this issue, the SE (Squeeze-and-Excitation) attention mechanism was incorporated into CSRNet to form the SE-CSRNet model. Rather than serving as a simple add-on, the SE module provides targeted optimization tailored to petal counting. It compresses spatial dimensions through global average pooling (Squeeze), captures global inter-channel dependencies, and adaptively reweights channels through fully connected layers [58]. This process emphasizes informative channels—such as those capturing petal contours and textures—while suppressing irrelevant ones, thereby improving specificity and efficiency in feature extraction. In petal counting scenarios, CSRNet's convolutional backbone often faces channel information redundancy, particularly when petal color resembles the background or illumination is uneven, causing the original feature maps to contain excessive irrelevant information and leading to density estimation bias. By introducing the SE mechanism, the model achieves channel-level “attention allocation”, for example enhancing responses to petal contours in dense inflorescences and strengthening edge recovery in occluded regions. Although visual inspection of the density maps in Fig. 3 may suggest that the spatial distribution of SE-CSRNet appears slightly different from the Ground Truth in some local regions, quantitative metrics including MAE and RMSE, which reflect the integrated accuracy over the entire density map, serve as the definitive measure of counting performance. SE-CSRNet achieves lower MAE and RMSE than the baseline CSRNet, indicating that it provides a more accurate total integral (i.e., petal count) and more reliable density estimation. The improved quantitative performance can be attributed to reduced noise interference and more precise estimation of density peaks, even if minor local spatial discrepancies are visually observed. These results confirm that SE-CSRNet yields lower MAE and RMSE values than the baseline CSRNet, particularly in dense and occluded scenarios. These findings are consistent with prior work showing the utility of SE modules in enhancing recognition and detection tasks [[59], [60], [61]].

Additionally, comparative tests with alternative attention mechanisms (CA, ECA, GAM, and SIM) further highlight the advantages SE for this application. While spatially focused modules such as CA and GAM provide stronger spatial attention, their computational burden limits real-time agricultural use. ECA is computationally efficient but models channel dependencies less effectively than SE. In contrast, SE strikes a balance between efficiency and accuracy, with its channel-level adaptivity aligning well with the spectral characteristics of petals. This property enhances generalization and scalability, offering a transferable strategy for counting similarly dense plant organs such as grains or leaves. Thus, SE-CSRNet not only advances automated floral morphology analysis but also broadens the scope of deep learning applications in plant phenotyping.

### 4.2 Improved petal count prediction accuracy

To further optimize numerical predictions, five common machine learning regression algorithms were tested on the density map outputs. Among them, random forest regression achieved the best overall performance across MSE, RMSE, MAE, and R^2^ metrics, reflecting its strong nonlinear fitting ability and generalization performance. As an ensemble method, random forest constructs multiple decision trees using bootstrap sampling and random feature subset selection, thereby reducing the variance and overfitting risk associated with individual trees [62]. In petal counting, density map integrals are often nonlinear due to petal overlap, deformities, and environmental noise. Linear regression cannot capture these nonlinearities, while single decision trees are prone to overfitting. Random forest, however, averages across multiple trees, mitigating noise sensitivity and balancing bias–variance trade-offs [63].

Furthermore, compared with support vector regression, random forest exhibits greater robustness to outliers, such as extreme occlusion, since noisy samples can be isolated within tree branches rather than influencing global optimization. Compared with gradient boosting, it avoids gradient instability and offers efficient parallel training, making it well-suited for small datasets [64]. This is consistent with previous applications of random forest in predicting agricultural phenotypic traits [[65], [66], [67]].

By integrating deep learning and machine learning in a two-stage pipeline, with CSRNet responsible for spatial feature extraction and random forest serving as the regression model, the proposed approach significantly improves both accuracy and stability. This result demonstrates the effectiveness of multi-model fusion strategies for complex agricultural trait estimation.

### 4.3 Application to cultivar evaluation under stress

The optimized SE-CSRNet model was further validated on two cut chrysanthemum cultivars (‘Yuka’ and ‘Huang Ping Pang’) to assess the effect of high-temperature stress on petal reduction. Although MAE and RMSE values were higher than in the controlled test set (13.95/17.42 for ‘Yuka’ and 29.88/35.32 for ‘Huang Ping Pang’), the model successfully performed accurate counting without having been trained on deformed petals. Moreover, statistical tests confirmed that high temperature significantly suppressed petal numbers, consistent with previous studies [11,68]. This application highlights two key points: first, the robustness and generalization of SE-CSRNet to previously unseen stress-induced deformations, and second, its potential as a quantitative tool for evaluating environmental stress tolerance in flower breeding programs. By enabling high-throughput screening of stress responses, the model provides practical support for breeding and production under climate variability.

Floral organ number is determined during early inflorescence development and is closely associated with meristem activity, organ primordium initiation rate, and hormonal regulation [[69], [70], [71]]. Elevated temperature has been reported to disrupt floral meristem determinacy and alter auxin and gibberellin signaling pathways, which in turn may reduce the initiation or proper differentiation of petal primordia [72,73]. In chrysanthemum, where petals are densely arranged and continuously differentiated during capitulum development, high-temperature stress may shorten the effective developmental window or impair organogenesis stability, ultimately leading to a reduction in final petal number [[74], [75], [76]]. Therefore, the decrease detected by SE-CSRNet is not merely a numerical change but likely reflects temperature-induced perturbations in floral developmental regulation.

Importantly, the proposed framework substantially improves phenotyping efficiency compared with manual counting. Conventional methods are typically low-throughput, making it difficult to capture population-level variation and environmental responses. In contrast, SE-CSRNet provides objective and scalable quantification of petal number, allowing simultaneous assessment of trait mean shifts and variability changes. In this study, the two cultivars showed differences not only in the absolute reduction in petal number under stress but also in their coefficients of variation, suggesting cultivar-dependent differences in developmental stability under high-temperature conditions. By transforming petal number into a reproducible quantitative trait, the proposed approach supports high-throughput evaluation of stress tolerance and cultivar robustness, demonstrating its broader value for digital floral phenotyping and climate-resilient breeding.

### 4.4 Limitations and future directions

Despite these advances, several limitations remain. First, the CSRNet and SE-CSRNet depend on single-view image inputs; in cases of severe overlap, prediction errors persist. Multi-view imaging or three-dimensional reconstruction could alleviate this issue. Although correlation analysis confirmed a strong relationship between visible and actual petal numbers, residual analysis indicates that prediction errors are not uniformly distributed across flower structures. Cultivars with extremely dense or compact inflorescences tend to show larger deviations due to severe occlusion and layered arrangements, where small local density estimation errors may accumulate during integration. In contrast, loosely arranged floral forms generally exhibit more stable predictions. These findings suggest that structural complexity and occlusion level influence model generalization and warrant stratified error evaluation in future work. Second, although SE enhances feature discrimination, its capacity may be insufficient for highly complex floral traits, such as irregular or extremely dense petals. Future work could explore hybrid attention mechanisms to improve adaptability. Third, most training data were collected under controlled conditions with uniform backgrounds and stable lighting. Field validation in natural environments with more complex conditions is essential to ensure robust generalization. Variations in illumination, background complexity, and natural field interference may alter feature distributions and affect both density estimation and regression calibration in the two-stage pipeline. Because the framework adopts a two-stage design, distributional shifts between training and deployment scenarios may influence the stability of the regression calibration stage. Therefore, cross-environment validation and domain adaptation strategies will be essential to enhance real-world robustness. Fourth, deformed petals were not explicitly annotated as a separate class. Introducing a “deformed-petal” subclass and applying multi-task learning to jointly output density maps and segmentation masks could improve interpretability and accuracy. In summary, this study demonstrates the effectiveness of combining deep learning and machine learning for robust petal number prediction in cut chrysanthemum. Future research should prioritize multi-view fusion, field generalization, and lightweight model deployment to enable scalable, real-world applications in flower breeding, production management, and digital agriculture.

## 5 Conclusion

This study developed and validated a hybrid framework integrating deep learning and machine learning for the automated extraction and prediction of petal numbers in cut chrysanthemum inflorescences. Correlation analysis demonstrated a strong positive linear relationship between visible and actual petal counts (r = 0.953, p < 0.0001), supporting the feasibility of estimating petal numbers from image-derived features. The proposed SE-CSRNet model, an improved variant of CSRNet integrated with a squeeze-and-excitation attention mechanism, reduced MAE and RMSE by 5.2% and 7.4%, respectively, and achieved improved test performance (MAE = 6.0621, RMSE = 9.3909) relative to the original CSRNet and other representative counting models (e.g., CANNet, MCNN). These results confirm that the incorporation of the SE attention mechanism effectively enhances feature representation and counting accuracy in dense floral organ counting tasks. For secondary regression fitting, five machine learning models were compared, among which the random forest model achieved the optimal performance (MAE = 4.2428, RMSE = 5.0621, R^2^ = 0.9667), further improving prediction accuracy and robustness. Application tests on two chrysanthemum cultivars under high-temperature stress conditions verified the practicality and adaptability of the proposed framework. Overall, the combination of the optimized SE-CSRNet detection model and machine learning regression provides a robust, accurate, and practically applicable solution for automated petal number estimation in cut chrysanthemums, offering technical support for intelligent high-throughput flower phenotyping. Notably, while correlation analysis demonstrates a high degree of linear consistency between estimated and ground-truth petal counts, we recognize that correlation alone is insufficient to ensure reliable recovery of absolute petal counts under varying occlusion levels and complex floral structures. Therefore, future work will be dedicated to exploring more effective solutions to address this limitation, including further optimization of model architecture and feature extraction strategies, with the goal of improving counting precision, generalization ability, and robustness under more challenging imaging conditions.

## Author contributions

Shanpeng Xu: Conceptualization, Data curation, Formal analysis, Investigation, Methodology, Software, Validation, Visualization, Writing – original draft, Writing – review & editing. Jingshan Lu: Conceptualization, Data curation, Formal analysis, Investigation, Methodology, Software, Validation, Visualization, Writing – original draft, Writing – review & editing. Lian Ding: Data curation, Investigation, Writing – original draft. Fei Zhang: Data curation, Investigation, Writing – original draft. Fadi Chen: Data curation, Investigation, Writing – original draft. Zhenxing Wang: Conceptualization, Methodology, Writing – original draft. Sumei Chen: Data curation, Investigation, Writing – original draft. Weimin Fang: Data curation, Investigation, Writing – original draft. Zhiyong Guan: Conceptualization, Resources, Writing – original draft, Writing – review & editing, Supervision, Project administration, Funding acquisition.

## Funding

This work was supported by the National Key Research and Development Program of China (2023YFD2300900), the National Natural Science Foundation of China (32302593). We are grateful to the editor and anonymous reviewers for their suggestions and comments, which significantly improved the quality of this paper.

## Declaration of competing interest

The authors declare that they have no known competing financial interests or personal relationships that could have appeared to influence the work reported in this paper.

## Data Availability

Some data will be available at this URL: https://github.com/qwsdfgz/petalscount. Other data are openly available from the corresponding author upon reasonable request.
