# Supplementary material for: Deep learning-driven automatic counting of petal number in cut chrysanthemum inflorescence
Source: Plant Phenomics. 2026 Jun 11;8(3):100238. doi: 10.1016/j.plaphe.2026.100238 (PMC13315742; doi:10.1016/j.plaphe.2026.100238)
Supplement: Multimedia component 1 [file mmc1.docx]

**Appendix**


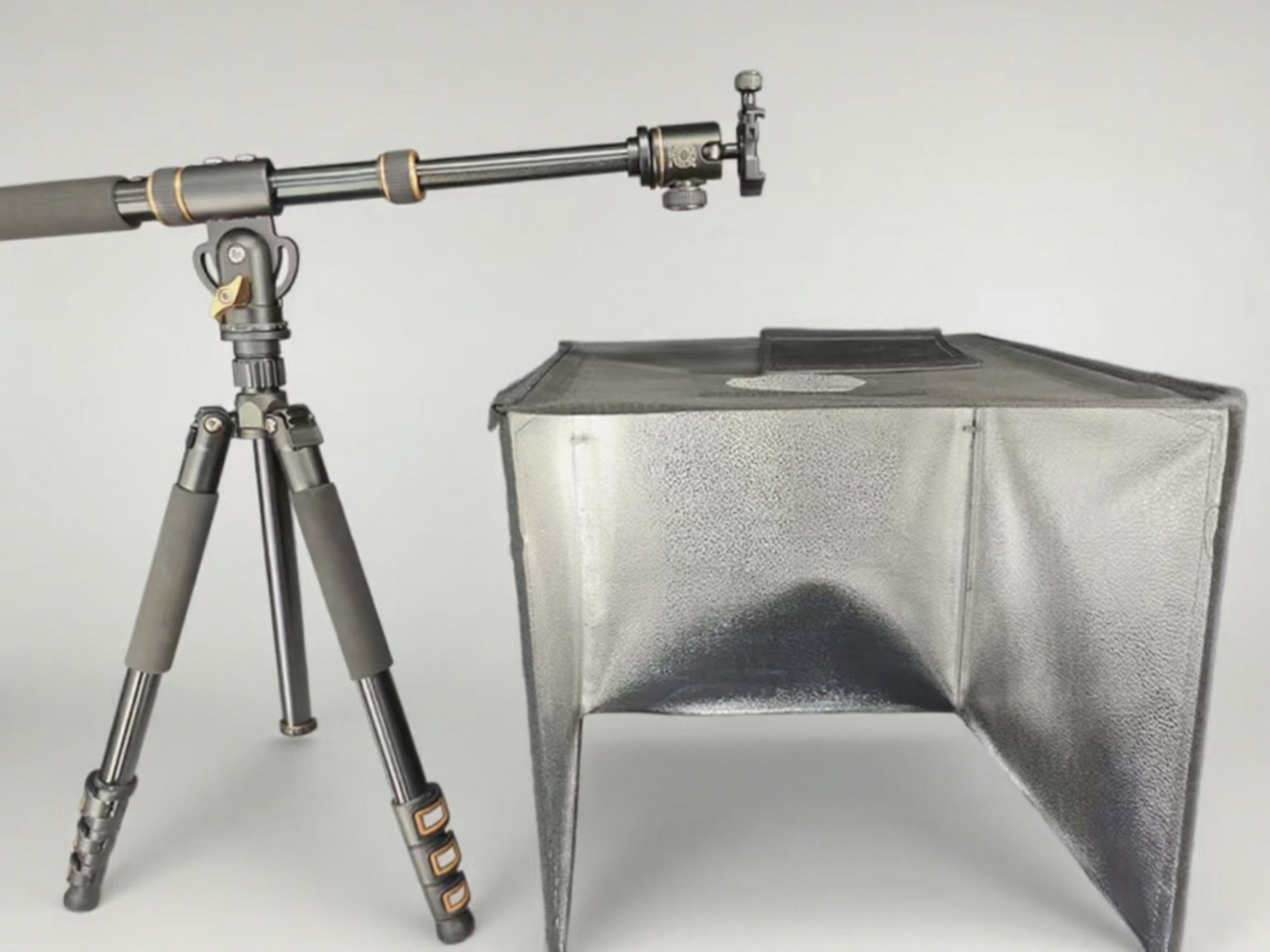


**Supplementary Fig.S1** Data collection diagram. This setup consists of a tripod with a horizontal boom arm for camera mounting, paired with a light-isolating enclosure. This configuration ensures controlled, uniform lighting and stable imaging conditions, which are critical for acquiring high-quality, consistent flower images to train and validate the deep learning models for automated petal counting.


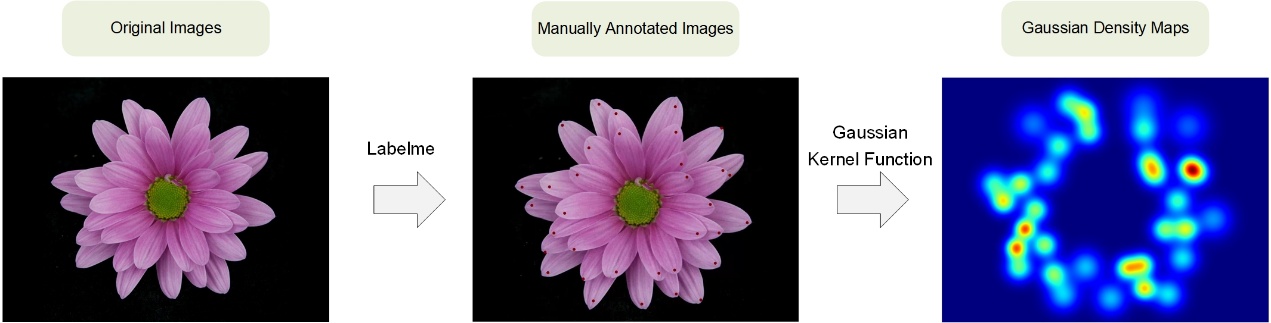


**Supplementary Fig.S2** Dataset preparation flowchart. This diagram illustrates the key steps in generating training data: starting from original flower images, manual annotations are created using Labelme to mark individual petal positions, which are then converted into continuous Gaussian density maps via a Gaussian kernel function. This process transforms discrete petal labels into a density representation suitable for training the CSRNet and SE-CSRNet models.


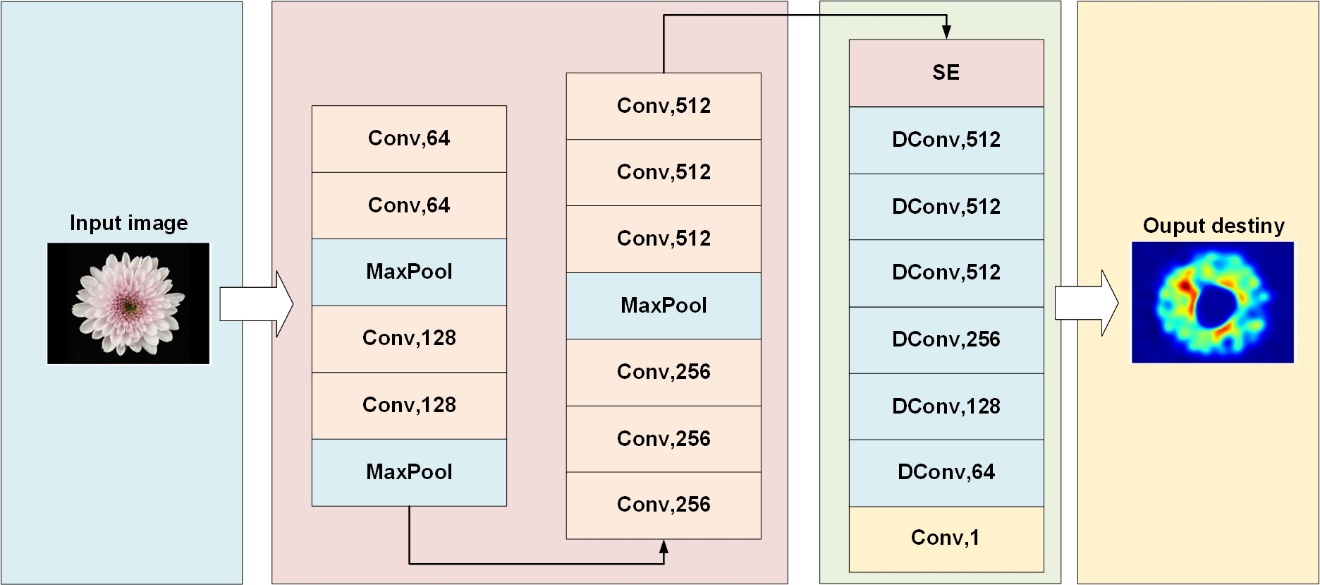


**Supplementary Fig.S3** CSRNet network structure diagram. Architecture of the SE-CSRNet model, which integrates a squeeze-and-excitation (SE) attention module into the CSRNet backbone to enhance feature recalibration for improved flower petal density map prediction.


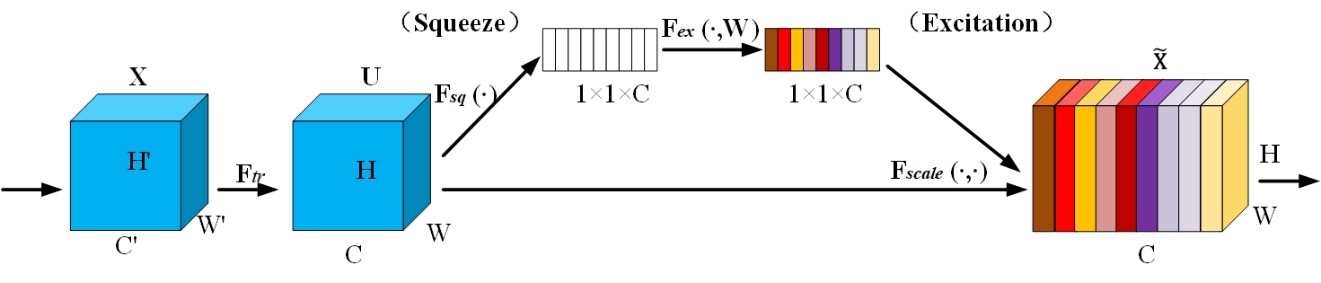


**Supplementary Fig.S4** SE Attention Module. This module performs channel-wise feature recalibration: it first squeezes global spatial information into channel descriptors, then excites adaptive channel weights to selectively emphasize informative features for enhanced model performance.


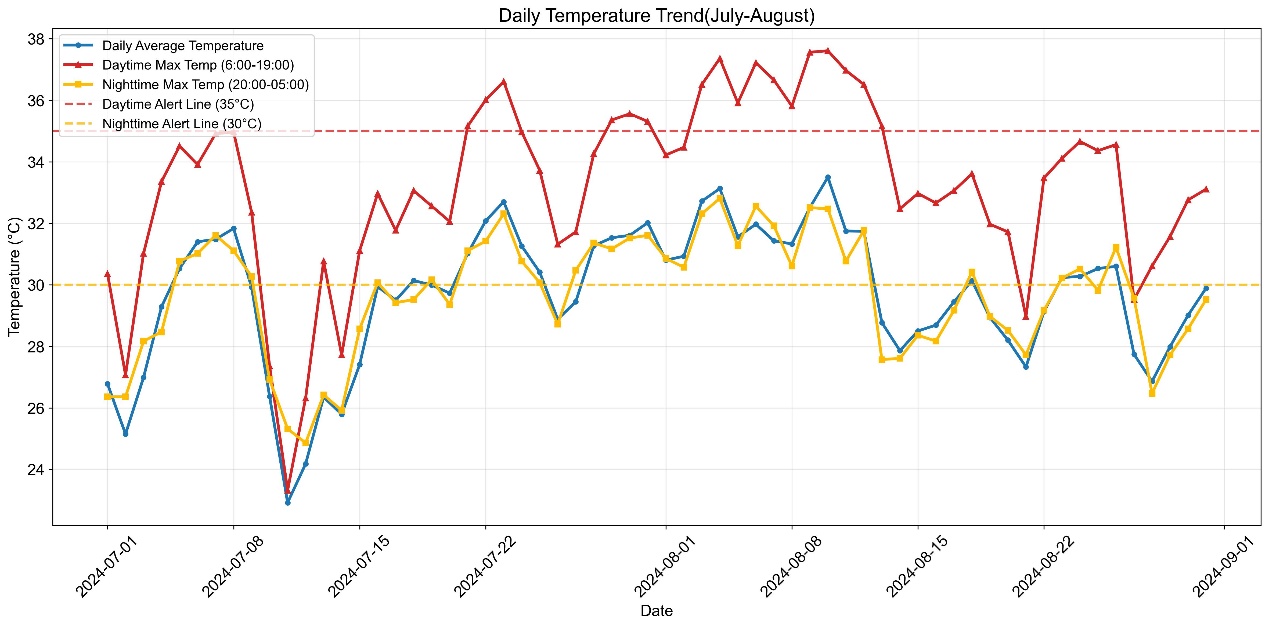


**Supplementary Fig.S5** Daily temperature trend (July–August). This figure shows daily average temperature (blue), daytime maximum temperature (red, 6:00–19:00), and nighttime maximum temperature (yellow, 20:00–05:00) over the experimental period. Red and yellow dashed lines indicate the 35°C daytime and 30°C nighttime temperature alert thresholds, respectively. The dataset reveals fluctuations in both mean and peak thermal conditions, providing a comprehensive overview of the daily thermal environment during July–August.


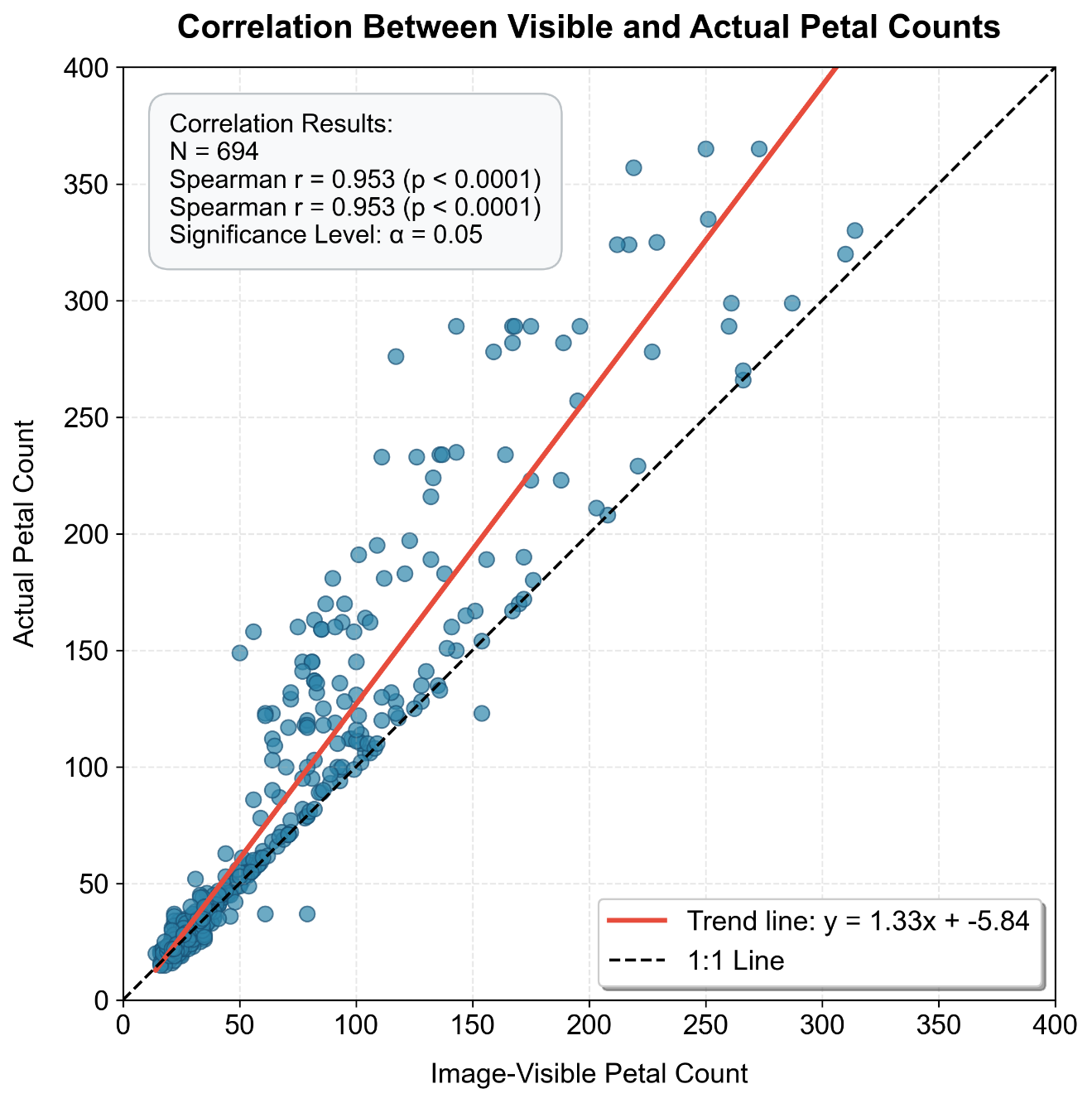


**Supplementary Fig.S6** Correlation analysis chart. This scatter plot shows a strong positive correlation (Spearman r = 0.953, p < 0.0001) between the number of petals visible in images and the actual petal counts (N = 694), with a trend line of y = 1.33x - 5.84, validating the feasibility of using image data for petal counting.


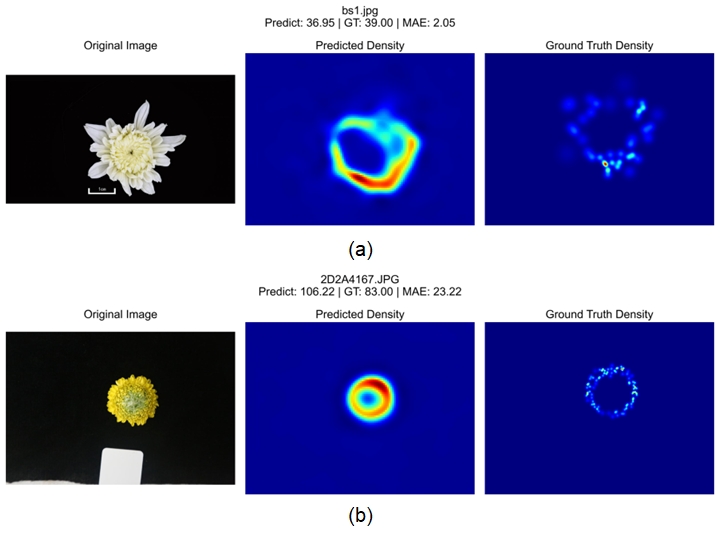


**Supplementary Fig.S7** Model detection result diagram. This figure presents two representative test cases (a, b), each showing the original flower image, the density map predicted by the model, and the ground truth density map, with corresponding predicted counts, ground truth counts, and MAE values provided for quantitative evaluation.

**Supplementary** **Tab.S1** Variety information table. This table summarizes the number, name, source, and type of all flower varieties, including both introduced multi-head cultivars and self-developed single-/multi-head varieties.

| Number | Variety Name | Source | Types |
| --- | --- | --- | --- |
| 1 | Vatican | Introduced | Multi-head |
| 2 | Nannong Bingyu | Self-developed | Multi-head |
| 3 | Nannong Yuhu | Self-developed | Multi-head |
| 4 | Nannong Xiaoyabai | Self-developed | Multi-head |
| 5 | Rossi white | Introduced | Multi-head |
| 6 | Q1-32 | Introduced | Multi-head |
| 7 | Neva | Introduced | Multi-head |
| 8 | Q1-49 | Introduced | Multi-head |
| 9 | Alts | Introduced | Multi-head |
| 10 | Nannong Xiaoqingxin | Self-developed | Multi-head |
| 11 | Nannong Binyun | Self-developed | Multi-head |
| 12 | Nannong Fangzhou | Self-developed | Multi-head |
| 13 | Nannong Bingquan | Self-developed | Multi-head |
| 14 | Nannong Bingjie | Self-developed | Multi-head |
| 15 | Nannong Kaige | Self-developed | Multi-head |
| 16 | Nannong Bingxue | Self-developed | Multi-head |
| 17 | Noa | Introduced | Multi-head |
| 18 | Nannong Zhishi | Self-developed | Multi-head |
| 19 | Nannong Biyun | Self-developed | Multi-head |
| 20 | Harley | Introduced | Multi-head |
| 21 | Nannong Xiangyang | Self-developed | Multi-head |
| 22 | Nannong Xiangcheng | Self-developed | Multi-head |
| 23 | Rossi | Introduced | Multi-head |
| 24 | Nannong Xinya | Self-developed | Multi-head |
| 25 | Q1-10 | Introduced | Multi-head |
| 26 | Nannong Xiangbinfen | Self-developed | Multi-head |
| 27 | Nannong Fenli | Self-developed | Multi-head |
| 28 | Nannong Fenyun | Self-developed | Multi-head |
| 29 | Nannong Binfen | Self-developed | Multi-head |
| 30 | Nannong Jiaren | Self-developed | Multi-head |
| 31 | Q1-112 | Introduced | Multi-head |
| 32 | Amadea | Introduced | Multi-head |
| 33 | Caesar Improved | Introduced | Multi-head |
| 34 | Grand Salmon | Introduced | Multi-head |
| 35 | Grand Splendid | Introduced | Multi-head |
| 36 | Nannong Fenye | Self-developed | Multi-head |
| 37 | Nannong Huaxianzi | Self-developed | Multi-head |
| 38 | Nannong Cuimei | Self-developed | Single-head |
| 39 | Q1-31 | Introduced | Multi-head |
| 40 | Q1-13 | Introduced | Multi-head |
| 41 | Q1-94 | Introduced | Multi-head |
| 42 | Nannong Hongyun | Self-developed | Multi-head |
| 43 | Nannong Huoshan | Self-developed | Multi-head |
| 44 | Q1-38 | Introduced | Multi-head |
| 45 | Harley Red | Introduced | Multi-head |
| 46 | Nannong Xiaojinhong | Self-developed | Multi-head |
| 47 | Nannong Hongqi | Self-developed | Multi-head |
| 48 | Marabou | Introduced | Multi-head |
| 49 | Nannong Hongrong | Self-developed | Multi-head |
| 50 | Nannong Hongque | Self-developed | Multi-head |
| 51 | Leopard | Introduced | Multi-head |
| 52 | Cinzia | Introduced | Multi-head |
| 53 | Cetebrate | Introduced | Multi-head |
| 54 | Q1-30 | Introduced | Multi-head |
| 55 | Q1-67 | Introduced | Multi-head |
| 56 | Nannong Danjincui | Self-developed | Multi-head |
| 57 | Nannong Jinguang | Self-developed | Multi-head |
| 58 | Nannong Fengshou | Self-developed | Multi-head |
| 59 | Nannong Huangjinjia | Self-developed | Multi-head |
| 60 | Nannong Huangying | Self-developed | Multi-head |
| 61 | Noa Yellow | Introduced | Multi-head |
| 62 | Q1-35 | Introduced | Multi-head |
| 63 | Nannong Xiaoningmeng | Self-developed | Multi-head |
| 64 | Nannong Jincan | Self-developed | Multi-head |
| 65 | Nannong Jinyan | Self-developed | Multi-head |
| 66 | Q1-64 | Introduced | Multi-head |
| 67 | Nannong Jinyi | Self-developed | Multi-head |
| 68 | Nannong Jinqi | Self-developed | Multi-head |
| 69 | Lisboa | Introduced | Multi-head |
| 70 | Q1-51 | Introduced | Multi-head |
| 71 | Nannong Xuanzi | Self-developed | Multi-head |
| 72 | Nannong Zimou | Self-developed | Multi-head |
| 73 | Nannong Xuanhong | Self-developed | Multi-head |
| 74 | Nannong Xunzhang | Self-developed | Multi-head |
| 75 | Nannong Caisidai | Self-developed | Multi-head |
| 76 | Q1-60 | Introduced | Multi-head |
| 77 | Handsome | Introduced | Multi-head |
| 78 | Nannong Sajin | Self-developed | Multi-head |
| 79 | Q1-5 | Introduced | Multi-head |
| 80 | Nannong Qicai | Self-developed | Multi-head |
| 81 | Greenlizard | Introduced | Multi-head |
| 82 | Nannong Ziyunying | Self-developed | Multi-head |
| 83 | Nannong Zirong | Self-developed | Multi-head |
| 84 | Nannong Qinfengche | Self-developed | Multi-head |
| 85 | Nannong Xiaoyudie | Self-developed | Multi-head |
| 86 | Nannong Baihudie | Self-developed | Multi-head |
| 87 | Nannong Fenfengche | Self-developed | Multi-head |
| 88 | Nannong Tianfengche | Self-developed | Multi-head |
| 89 | Nannong Xiufengche | Self-developed | Multi-head |
| 90 | Nannong Yafengche | Self-developed | Multi-head |
| 91 | Nannong Jifengche | Self-developed | Multi-head |
| 92 | Nannong Zhanfengche | Self-developed | Multi-head |
| 93 | Nannong Huofengche | Self-developed | Multi-head |
| 94 | Q5-13 | Introduced | Multi-head |
| 95 | Nannong Jinhudie | Self-developed | Multi-head |
| 96 | Roma Festa | Introduced | Multi-head |
| 97 | Q5-2 | Introduced | Multi-head |
| 98 | Nannong Yingluo | Self-developed | Multi-head |
| 99 | Q5-6 | Introduced | Multi-head |
| 100 | Nannong Qingfengche | Self-developed | Multi-head |
| 101 | Nannong Lifengche | Self-developed | Multi-head |
| 102 | Nannong Xiafengche | Self-developed | Multi-head |
| 103 | Nannong Chenfengche | Self-developed | Multi-head |
| 104 | Nannong Wufengche | Self-developed | Multi-head |
| 105 | Nannong Qiaofengche | Self-developed | Multi-head |
| 106 | Nannong Lvhudie | Self-developed | Multi-head |
| 107 | Nannong Ziyunjian | Self-developed | Multi-head |
| 108 | Nannong Meifengche | Self-developed | Multi-head |
| 109 | Q1-11 | Introduced | Multi-head |
| 110 | Nannong Yuzhu | Self-developed | Multi-head |
| 111 | Feeling White | Introduced | Single-head |
| 112 | Nannong Cuiyuzhu | Self-developed | Multi-head |
| 113 | Nannong Yukou | Self-developed | Multi-head |
| 114 | Jeanny Orange | Introduced | Multi-head |
| 115 | Pinky Rock Dark | Introduced | Multi-head |
| 116 | Jeanne Pink | Introduced | Multi-head |
| 117 | Feeling Sunny | Introduced | Single-head |
| 118 | Nannong Jinkouzi | Self-developed | Multi-head |
| 119 | Snazzy | Introduced | Multi-head |
| 120 | Cayman | Introduced | Multi-head |
| 121 | Nannong Jinzhu | Self-developed | Multi-head |
| 122 | Q3-10 | Introduced | Multi-head |
| 123 | Marimo | Introduced | Multi-head |
| 124 | Feeling Green Dark | Introduced | Single-head |
| 125 | Nannong Luyi | Self-developed | Multi-head |
| 126 | Nannong Doulu | Self-developed | Multi-head |
| 127 | Nannong Xuanlu | Self-developed | Multi-head |
| 128 | Nannong Lvyuan | Self-developed | Multi-head |
| 129 | Nannong Cuipingpang | Self-developed | Multi-head |
| 130 | Olive | Introduced | Multi-head |
| 131 | Country | Introduced | Multi-head |
| 132 | Nannong Lvxing | Self-developed | Multi-head |
| 133 | Nannong Yurong | Self-developed | Multi-head |
| 134 | Nannong Zizhu | Self-developed | Multi-head |
| 135 | Q1-48 | Introduced | Multi-head |
| 136 | Nannong Daiyue | Self-developed | Multi-head |
| 137 | Nannong Luyun | Self-developed | Multi-head |
| 138 | Nannong Songyun | Self-developed | Multi-head |
| 139 | Nannong Hengyue | Self-developed | Multi-head |
| 140 | Nannong Qingquan | Self-developed | Multi-head |
| 141 | Nannong Yunyue | Self-developed | Multi-head |
| 142 | DanteSalmon | Introduced | Multi-head |
| 143 | Nannong Jintai | Self-developed | Multi-head |
| 144 | Nannong Songhui | Self-developed | Multi-head |
| 145 | Candor Pink | Introduced | Multi-head |
| 146 | Nannong Xiaofenlu | Self-developed | Multi-head |
| 147 | Nannong Fenlu | Self-developed | Multi-head |
| 148 | Q4-35 | Introduced | Multi-head |
| 149 | Tucana | Introduced | Multi-head |
| 150 | Nannong Liya | Self-developed | Multi-head |
| 151 | Nannong Hengxiu | Self-developed | Multi-head |
| 152 | Nannong Hengyun | Self-developed | Multi-head |
| 153 | Nannong Luxia | Self-developed | Multi-head |
| 154 | Lollipop | Introduced | Multi-head |
| 155 | Royce Lovely | Introduced | Multi-head |
| 156 | Nannong Daifen | Self-developed | Multi-head |
| 157 | Nannong Hengchun | Self-developed | Multi-head |
| 158 | Nannong Daihua | Self-developed | Multi-head |
| 159 | Nannong Daili | Self-developed | Multi-head |
| 160 | Nannong Luchun | Self-developed | Multi-head |
| 161 | Urban | Introduced | Multi-head |
| 162 | Dante Red | Introduced | Multi-head |
| 163 | Nannong Luhuo | Self-developed | Multi-head |
| 164 | Nannong Qiulu | Self-developed | Multi-head |
| 165 | Q4-52 | Introduced | Multi-head |
| 166 | Nannong Hengxing | Self-developed | Multi-head |
| 167 | Nannong Taihui | Self-developed | Multi-head |
| 168 | Nannong Songyao | Self-developed | Multi-head |
| 169 | Nannong Hengyang | Self-developed | Multi-head |
| 170 | Ibis Sunny | Introduced | Multi-head |
| 171 | Nannong Jinsong | Self-developed | Multi-head |
| 172 | Nannong Jinlu | Self-developed | Multi-head |
| 173 | Nannong Hengyao | Self-developed | Multi-head |
| 174 | Euro Sunny | Introduced | Multi-head |
| 175 | Q4-51 | Introduced | Multi-head |
| 176 | Nannong Henghui | Self-developed | Multi-head |
| 177 | Nannong Chihuang | Self-developed | Multi-head |
| 178 | Pip | Introduced | Single-head |
| 179 | Q4-3 | Introduced | Multi-head |
| 180 | Nannong Songrong | Self-developed | Multi-head |
| 181 | Q4-32 | Introduced | Multi-head |
| 182 | Nannong Lvmeigui | Self-developed | Multi-head |
| 183 | Nannong Lvsheyao | Self-developed | Multi-head |
| 184 | Nannong Emei | Self-developed | Multi-head |
| 185 | Nannong Zidan | Self-developed | Multi-head |
| 186 | Dante Purple | Introduced | Multi-head |
| 187 | Nannong Songzi | Self-developed | Multi-head |
| 188 | Nannong Xiaosongzi | Self-developed | Multi-head |
| 189 | Quinty | Introduced | Multi-head |
| 190 | Q5-11 | Introduced | Single-head |
| 191 | Q5-20 | Introduced | Single-head |
| 192 | Nannong Gusheng | Self-developed | Multi-head |
| 193 | Nannong Xiaolibai | Self-developed | Multi-head |
| 194 | Nannong Yunque | Self-developed | Multi-head |
| 195 | Nannong Libai | Self-developed | Multi-head |
| 196 | Nannong Yupan | Self-developed | Multi-head |
| 197 | Nannong Xingkong | Self-developed | Multi-head |
| 198 | Radost | Introduced | Multi-head |
| 199 | Q2-27 | Introduced | Multi-head |
| 200 | Nannong Licui | Self-developed | Multi-head |
| 201 | Nannong Yuegui | Self-developed | Multi-head |
| 202 | Nannong Xuefeng | Self-developed | Multi-head |
| 203 | Nannong Yinshan | Self-developed | Multi-head |
| 204 | Nannong Xingyun | Self-developed | Multi-head |
| 205 | Monalisa White | Introduced | Multi-head |
| 206 | Puma White | Introduced | Multi-head |
| 207 | Nannong Xiaocuijie | Self-developed | Multi-head |
| 208 | Nannong Qingyu | Self-developed | Multi-head |
| 209 | Nannong Xiaolicui | Self-developed | Multi-head |
| 210 | Nannong Lixue | Self-developed | Multi-head |
| 211 | Nannong Liyun | Self-developed | Multi-head |
| 212 | Nannong Nuanyang | Self-developed | Multi-head |
| 213 | Nannong Chengying | Self-developed | Multi-head |
| 214 | Nannong Lipo | Self-developed | Multi-head |
| 215 | Nannong Licheng | Self-developed | Multi-head |
| 216 | Nannong Xixia | Self-developed | Multi-head |
| 217 | Q2-17 | Introduced | Multi-head |
| 218 | Finch | Introduced | Multi-head |
| 219 | Nannong Luoying | Self-developed | Multi-head |
| 220 | Nannong Fencui | Self-developed | Multi-head |
| 221 | Nannong Feipeng | Self-developed | Multi-head |
| 222 | Nannong Xiaolifen | Self-developed | Multi-head |
| 223 | Nannong Fenyu | Self-developed | Multi-head |
| 224 | Nannong Limei | Self-developed | Multi-head |
| 225 | Nannong Fenying | Self-developed | Multi-head |
| 226 | Nannong Yali | Self-developed | Multi-head |
| 227 | Nannong Fenkui | Self-developed | Multi-head |
| 228 | Nannong Xingyu | Self-developed | Multi-head |
| 229 | Nannong Xingmeng | Self-developed | Multi-head |
| 230 | Monalisa Pink | Introduced | Multi-head |
| 231 | Nannong Libin | Self-developed | Multi-head |
| 232 | Nannong Luoyan | Self-developed | Multi-head |
| 233 | Q2-21 | Introduced | Multi-head |
| 234 | Nannong Xiaofenkui | Self-developed | Multi-head |
| 235 | Nannong Menglu | Self-developed | Multi-head |
| 236 | Nannong Feiyu | Self-developed | Multi-head |
| 237 | Nannong Meifen | Self-developed | Multi-head |
| 238 | Nannong Lifen | Self-developed | Multi-head |
| 239 | Nannong Xiaocaomei | Self-developed | Multi-head |
| 240 | Monalisa Rosy | Introduced | Multi-head |
| 241 | Samos | Introduced | Multi-head |
| 242 | Samos Dark | Introduced | Multi-head |
| 243 | Nannong Shuanggui | Self-developed | Multi-head |
| 244 | Nannong Xiayi | Self-developed | Multi-head |
| 245 | Nannong Fenyan | Self-developed | Multi-head |
| 246 | Nannong Caiyu | Self-developed | Multi-head |
| 247 | Nannong Yanzhiyu | Self-developed | Multi-head |
| 248 | Q2-20 | Introduced | Multi-head |
| 249 | Nannong Lifeng | Self-developed | Multi-head |
| 250 | Nannong Hongjiao | Self-developed | Multi-head |
| 251 | Nannong Lihong | Self-developed | Multi-head |
| 252 | Monalisa Currant | Introduced | Multi-head |
| 253 | Puma Sunny | Introduced | Multi-head |
| 254 | Sunny | Introduced | Single-head |
| 255 | Nannong Huangli | Self-developed | Multi-head |
| 256 | Nannong Lihuang | Self-developed | Multi-head |
| 257 | Nannong Xiaojinxing | Self-developed | Multi-head |
| 258 | Monalisa Yellow | Introduced | Multi-head |
| 259 | Nannong Bingqilin | Self-developed | Multi-head |
| 260 | Nannong Xiaguang | Self-developed | Multi-head |
| 261 | Nannong Chifeng | Self-developed | Multi-head |
| 262 | Monalis Cream | Introduced | Multi-head |
| 263 | Nannong Xuri | Self-developed | Multi-head |
| 264 | Flashy | Introduced | Multi-head |
| 265 | Nannong Bianlian | Self-developed | Multi-head |
| 266 | Nannong Feiyan | Self-developed | Multi-head |
| 267 | Nannong Hualian | Self-developed | Multi-head |
| 268 | Nannong Liyan | Self-developed | Multi-head |
| 269 | Nannong Yanzhimei | Self-developed | Multi-head |
| 270 | Nannong Yamei | Self-developed | Multi-head |
| 271 | Nannong Dahualian | Self-developed | Multi-head |
| 272 | Nannong Xiaoguilian | Self-developed | Multi-head |
| 273 | Nannong Chenxia | Self-developed | Multi-head |
| 274 | Nannong Cuiyu | Self-developed | Multi-head |
| 275 | Nannong Xuankui | Self-developed | Multi-head |
| 276 | Nannong Guilian | Self-developed | Multi-head |
| 277 | Nannong Jinhong | Self-developed | Multi-head |
| 278 | Nannong Ziyu | Self-developed | Multi-head |
| 279 | Nannong Zicui | Self-developed | Multi-head |
| 280 | Nannong Liebo | Self-developed | Multi-head |
| 281 | Nannong Zixia | Self-developed | Multi-head |
| 282 | Nannong Yanzi | Self-developed | Multi-head |
| 283 | Nannong Zimei | Self-developed | Multi-head |

**Supplementary** **Tab.S2** Deep learning machine parameters. This table lists the key hardware and software configurations, including operating system, CPU, graphics card, and RAM.

| Category | Details |
| --- | --- |
| Operating System | Windows11 |
| CPU | Intel(R) Xeon(R) Gold 6240R CPU @ 2.40GHz |
| Graphics Card Model | NVIDIA RTX4090 |
| RAM | 128 GB |

**Supplementary Tab.S3** Performance comparison of different attention modules on the validation set. Performance comparison of CSRNet with different attention modules on the validation set, evaluated by MAE and RMSE.

| Model | MAE | RMSE |
| --- | --- | --- |
| CSRNet | 5.777 | 9.837 |
| CA-CSRNet | 6.319 | 13.946 |
| ECA-CSRNet | 5.580 | 9.506 |
| GAM-CSRNet | 5.857 | 10.856 |
| SE-CSRNet | **5.475** | **9.102** |
| SIM-CSRNet | 6.248 | 11.183 |

**Supplementary Tab.S4** Performance comparison of different models on the validation set. Performance comparison of different counting models on the validation set in terms of MAE and RMSE.

| Model | MAE | RMSE |
| --- | --- | --- |
| CSRNet | 5.777 | 9.837 |
| CANNet | 15.429 | 26.149 |
| MCNN | 79.235 | 83.99 |
| SASNet | 30.485 | 49.829 |
| SE-CSRNet | **5.475** | **9.102** |

**Supplementary Tab.S5** Performance comparison before and after model improvement on the independent test set. Performance comparison of original and improved models on the independent test set using MAE and RMSE.

| Model | MAE | RMSE | Parameters(M) | GFLOPs | Inference speed(ms/P) |
| --- | --- | --- | --- | --- | --- |
| CSRNet | 6.7308 | 12.5178 | 16.26 | 132.2 | 17.45 |
| SE-CSRNet | 6.0621 | 9.3909 | 16.29 | 132.2 | 17.65 |
| CANNet | 16.961 | 30.398 | 18.1 | 140.05 | 18.01 |
| MCNN | 88.367 | 97.235 | 0.19 | 25.248 | 4.6 |
| SASNet | 35.146 | 60.145 | 38.9 | 698.95 | 92.3 |

**Supplementary Tab.S6** Comparison of fitting results among different models. This table presents the fitting results of five regression models, evaluated using four metrics: MSE, RMSE, MAE, and R².

| Model | MSE | RMSE | MAE | R^2^ |
| --- | --- | --- | --- | --- |
| Linear Regression | 37.0696 | 6.0885 | 4.6333 | 0.9519 |
| Lasso Regression | 37.0827 | 6.0896 | 4.6341 | 0.9518 |
| SVR | 121.3231 | 11.0147 | 7.5663 | 0.8424 |
| KNN | 118.5570 | 10.8884 | 6.1675 | 0.8460 |
| Random Forest Regression | **25.6249** | **5.0621** | **4.2428** | **0.9667** |

**Supplementary** **Tab.S7** Model processing results. Performance of the model on different flower varieties, evaluated by MAE and RMSE.

| Variety Name | MAE | RMSE |
| --- | --- | --- |
| ‘Yuka’ | 13.95 | 17.42 |
| ‘Huang Ping Pang’ | 29.88 | 35.32 |

**Supplementary** **Tab.S8** Feature difference table. This table shows the sample size, mean petal count per plant, standard deviation, and coefficient of variation for two cultivars under different temperature conditions.

| Cultivar | Group | Sample Size (n) | Mean (petals/plant) | Standard Deviation | Coefficient of Variation(%) |
| --- | --- | --- | --- | --- | --- |
| ‘Yuka’ | Control | 11 | 84.45 | 24.71 | 29.26 |
|  | High-Temperature | 11 | 40.00 | 8.04 | 20.09 |
| ‘Huang Ping Pang’ | Control | 47 | 388.43 | 25.07 | 6.45 |
|  | High-Temperature | 47 | 155.49 | 47.54 | 30.57 |

**Supplementary** **Tab.S9** Independent sample t-test result. Results of independent sample t-test between control and high-temperature groups.

| Cultivar | Test Type | Statistic | p-value | Homogeneity of Variance | t-test Type | Between-Group Significance |
| --- | --- | --- | --- | --- | --- | --- |
| ‘Yuka’ | Levene’s test | 3.759 | 0.067 | Homogeneous | Standard t-test | Highly significant（p<0.001） |
|  | Independent samples t-test | 5.674 | <0.001 |  |  |  |
| ‘Huang Ping Pang’ | Levene’s test | 17.885 | <0.001 | Heterogeneous | Welch’s t-test | Highly significant（p<0.001） |
|  | Independent samples t-test | 29.713 | <0.001 |  |  |  |
